# A Comparative Sound Intensity Method for Measuring the Increase in Sound Insulation of Small Acoustic Metamaterial Samples

**DOI:** 10.3390/s26103242

**Published:** 2026-05-20

**Authors:** Polaczek Agata, Baruch-Mazur Katarzyna, Ziarko Bartłomiej, Lewińska-Maresca Mirosława, Młynarczyk Dorota, Dusza Katarzyna

**Affiliations:** 1 Faculty of Civil Engineering, Cracow University of Technology, 31-155 Cracow, Poland; katarzyna.baruch@pk.edu.pl (B.-M.K.); bartlomiej.ziarko@pk.edu.pl (Z.B.); miroslawa.maresca@pk.edu.pl (L.-M.M.); dorota.mlynarczyk@pk.edu.pl (M.D.); katarzyna.dusza@pk.edu.pl (D.K.); 2 Agata Polaczek SZA Pracownia Akustyczna, 30-148 Cracow, Poland; 3 Andrzej Kĺosak archAKUSTIK, 31-143 Cracow, Poland; 4 NOVA Optical Infrared Instrumentation Group, ASTRON, 7991 PD Dwingeloo, The Netherlands; 5 Department of Mechanics and Vibroacustics, Faculty of Mechanical Engineering and Robotics, AGH University of Krakow, 30-059 Cracow, Poland

**Keywords:** transmission loss, scale measurements, sound insulation, acoustic metamaterials, insertion loss, sound intensity measurement, small-scale measurements

## Abstract

This paper presents a method for determining the reduction in noise transmission provided by small samples of acoustic metamaterials, based on comparative sound intensity measurements. The proposed approach offers an alternative to conventional laboratory methods that require large specimens and controlled acoustic conditions, which limit the rapid testing of prototypes. As part of this study, a mobile and modular measurement setup was developed in the form of a cubic enclosure with replaceable panels, enabling experiments to be conducted under near-real conditions. The measurement methodology is based on determining the difference in sound intensity level, $\Delta L_{I}$, between a reference configuration and a configuration with an installed metamaterial lining, which allows for the direct evaluation of the increase in sound insulation of the tested partition. To verify the method, a locally resonant metamaterial structure was designed and numerically tuned to a frequency of approximately 460 Hz. Physical samples were then fabricated using 3D printing technology and experimentally tested for two variants of base partitions with different sound insulation performance. The obtained results showed a clear noise transmission reduction in the vicinity of the tuning frequency, reaching approximately 17 dB for the partition with a lower baseline sound insulation and approximately 10 dB for the more insulating partition. A dependence of the metamaterial effectiveness on the properties of the base partition was also observed. The results confirm that the proposed method enables a reliable assessment of the influence of metamaterial structures on the noise transmission reduction of partitions using small samples and a simplified measurement setup.

## 1. Introduction

Methods for assessing the increase in sound insulation provided by acoustic materials are generally based on attenuation indicators or emitted acoustic power, determined under specific acoustic conditions, on dedicated test setups, and for predefined specimen sizes. These requirements make sample preparation and measurements time-consuming, which is a major limitation for rapid testing of prototypes, especially for metamaterials, whose complex elements are costly and time-consuming to manufacture. To address this limitation, the present study proposes a small, mobile, demountable, and readily accessible measurement setup. It enables the installation of separate enclosure walls and the attachment of additional structures. The method is demonstrated for additional metamaterial linings mounted on lightweight gypsum plasterboard partitions, although other lightweight homogeneous or multilayer base walls, such as wood, aluminum, or metamaterial-based panels, may also be used. For metamaterials, transmission loss (TL) measurements in an impedance tube, performed in accordance with ISO 10534-2, [1] are commonly used. This approach has been applied in studies comparing experimental and numerical results for circular samples fitted to the tube walls [2,3] or embedded in holders matched to the tube geometry [4]. Although this method requires only small specimens, it is limited in frequency range, is sensitive to mounting conditions, and represents only normal sound-wave incidence. Therefore, the sound reduction index, *R*, determined according to ISO 10140 [5] in coupled reverberation chambers, is widely used to assess the acoustic performance of partitions under more representative incidence conditions. However, this method requires laboratory facilities and relatively large specimens mounted between two chambers. Examples involving metamaterial structures include a 0.6 × 0.6 m membrane-based system [6] and a 1.14 × 1.14 m layered membrane specimen used to determine an increase in sound insulation [7].

Sound insulation measurements may also be performed using a sound intensity probe in large acoustic chambers, in accordance with ISO 15186-1 [8]. In such arrangements, the source room is typically reverberant, whereas the receiving room may be either reverberant or semi-anechoic. Intensity-based measurements with a reverberant receiving room have been reported for resonant metamaterials intended for aircraft wall applications [9]. More commonly, intensity-probe measurements are carried out in semi-anechoic chambers. Examples include sandwich metamaterials with an extended internal lattice tested on a 1.25 × 1.50 m specimen [10], acoustic black hole structures with dimensions of 0.85 × 0.86 m [11], steel-sheet metamaterial arrays tested on a 0.80 × 0.80 m specimen [12], and measurements over a 1.2 × 1.2 m opening compared with impedance-tube-based simulations [13,14]. Despite their usefulness, these methods require large specimens, often exceeding 1 $m^{2}$, which increases fabrication costs and complicates prototype development. Mounting large or mechanically fragile samples may also be problematic and may require additional protection or stiffening. Moreover, the need for specialized laboratory facilities and time-consuming specimen installation limits measurement efficiency and rapid prototype verification.

An alternative to testing such large specimens is the use of coupled reverberation chambers on a reduced scale. An example of such a setup is a 1:8-scale reverberation chamber suite [15], implemented in full compliance with ISO 10140 and capable of measuring specimens as small as 87.5 mm × 87.5 mm. Such setups have been used to determine the sound insulation of homogeneous materials, including steel, gypsum plasterboard, and wood-based panels [16,17,18], as well as simple multilayer systems [18]. A study on resonant metamaterials was conducted using a setup that partially adopted the concept of scaled chambers, with only the source room being reduced in size [19]. This configuration made it possible to measure the sound reduction index *R* according to ISO 10140 while reducing the specimen size to 450 × 550 mm and still maintaining controlled conditions in the receiving reverberation room. Although the selected setup did not satisfy all the requirements of ISO 10140, the cited study emphasized the advantage of reduced specimen dimensions from the perspective of prototype fabrication. Although measurements in a small reverberation chamber are more convenient and allow controlled testing, and specimen installation is faster than in a suite of large chambers, they still do not provide a fully practical assessment of sound insulation from the perspective of engineering applications because of the nature of the acoustic field in the receiving chamber.

A more practical alternative is to eliminate the receiving chamber and record the receiving-side response in a near-real environment using a sound intensity probe. This approach provides easier access to the specimens, reduces the need for strict control of the receiving field, and better reflects practical operating conditions. One example is the KU Leuven Soundbox [20,21], in which a concrete reverberation chamber with a front aluminum wall is used along with external scanning by a sound intensity probe. The setup includes a 420 × 594 mm specimen opening and satisfies diffuse-field conditions in the source chamber from 1600 Hz upwards. However, its concrete construction makes the system heavy. A similar reduced-scale approach was used to investigate the TL of membrane metamaterials in an anechoic chamber, with a cutoff frequency of 315 Hz and a 0.241 m × 0.241 m measurement window [22].

However, reduced-scale setups do not always satisfy the scaled requirements of standards developed for full-size chamber suites. For instance, the KU Leuven Soundbox is not fully adapted for TL measurements [20]; therefore, insertion loss (IL) is determined instead, as the difference between sound intensity levels measured at the outer surface of the specimen with and without the specimen. Similar comparative indicators have been reported as differences between acoustic power levels [20,23,24] or sound intensity levels [9,12] measured on the receiving side before and after the introduction of the tested element. This concept can also be applied to enclosure sound power measurements, where comparing a device with and without an enclosure enables the assessment of its sound-insulating effect under operating conditions, which is particularly useful for prototypes requiring long 3D-printing times. A metamaterial enclosure based on a similar concept was previously presented in [25], where TL was determined from the logarithmic difference between acoustic power levels. In that study, the enclosure surrounded the source on five sides, while the source was placed on a flat surface. However, the work did not address the increase in sound insulation obtained by installing a metamaterial on a standard enclosure.

IL based on acoustic power measurements can also be estimated from sound pressure levels measured over a surface surrounding the source, in accordance with ISO 3746 [26], although this requires environmental corrections. This approach was used in the AGH measurement setup [27,28,29], which consists of a demountable cubic enclosure with removable walls fitted with homogeneous materials such as steel, aluminum, plexiglass, and mineral wool. These studies focused mainly on validating computational models and clearly distinguished between TL and IL. Another demountable setup developed in Poland [30] used a heavy welded-steel enclosure to analyze changes in the vibration characteristics of a homogeneous steel plate with additional passive mass. Although this confirms the possibility of modifying partition behavior by structural changes, the study was limited to vibration analysis and did not consider metamaterial systems or determine sound insulation improvement.

Mobile and demountable measurement setups reported so far have mainly been validated against numerical models for homogeneous partitions and have typically used sound pressure level measurements with a sound level meter. They have not generally addressed structures as complex as metamaterials. One exception is the measurement of a membrane metamaterial in a semi-anechoic chamber using a small enclosure with an internal source [31]. However, resonances within the enclosure led to discrepancies between numerical transmission loss results and measurements. This indicates that determining TL in such systems requires full validation, as in Kosala’s approach [28]. In contrast, an indicator describing the increase in sound insulation can be determined comparatively and is less dependent on the detailed characteristics of the acoustic field inside the enclosure.

In the literature on resonant metamaterials, there is a trend toward comparing the sound insulation performance of a metamaterial composed of a plate and resonators with that of the plate alone or of a plate with equivalent mass [19,20,24]. In such cases, the determined indicator of increased sound insulation refers to the metamaterial structure itself, with the aim of tuning the resonant elements so that they interact properly with the thin plate. Even the attachment of another type of metamaterial, based on a wooden lattice, was implemented on an aluminium plate with a thickness of 2.35 mm [14]. However, there are still very few clear studies on the increase in TL for a complete metamaterial installed on a lightweight base panel, for example, gypsum plasterboard, relative to the insulation of the base panel alone under actual operating conditions. Laboratory tests [32] that considered a similar approach were based on initial enclosures made of thin plastic or steel, to the inner side of which the metamaterial was attached, while the entire experiment was conducted in an anechoic chamber. Despite the growing interest in applying acoustic metamaterials to the built environment and transportation, as evidenced by recent review papers [33,34] and the development of modular metamaterial designs [35,36,37], studies investigating their performance on realistic lightweight partitions under actual operating conditions remain limited.

In this work, an approach is proposed that combines the advantages of methods previously treated separately—namely, the use of a small, mobile, durable, and demountable measurement enclosure with an omnidirectional source inside (features found in the setups described in [28,29,30,31]), together with a procedure for measuring the sound insulation of metamaterials using a sound intensity probe in an environment that does not require special acoustic conditions [20,21]. Moreover, a key aspect of the proposed approach is the possibility of determining the increase in sound insulation for a complete resonant metamaterial system mounted on a lightweight gypsum plasterboard base panel, rather than only the sound insulation of the metamaterial structure itself or the increase in sound insulation obtained by attaching resonant metamaterial elements to the thin plate forming part of that same metamaterial. This approach introduces an element of assessment that is missing in the literature and constitutes an important step toward the practical application of metamaterials in engineering solutions.

## 2. Measurement Setup

### 2.1. Measurement Box

The measurement setup was developed for comparative investigations of the increase in sound insulation provided by small samples of metamaterial structures. The key features of the setup are mobility, modularity, and adaptability to changing experimental conditions. Its main component is a specially designed enclosure, hereinafter referred to as the measurement box, which serves as the source chamber.

**Figure 1:**
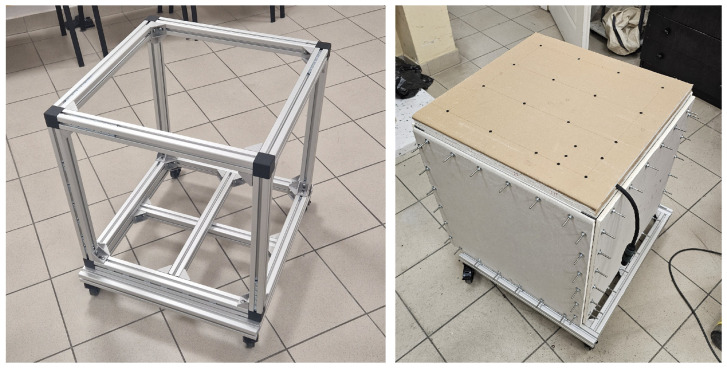
Aluminium frame of the measurement box (**left**) and the inverted frame enclosed with gypsum plasterboards mounted on threaded rods, with a cable gland for the loudspeaker cable (**right**).

The structure of the setup is based on an aluminium frame made of profiles with a cross-section of 2×2cm, forming the skeleton of the entire enclosure (Figure 1). The frame was mounted on a platform equipped with four swivel casters, which ensures the mobility of the setup and facilitates its transport between different measurement locations. The measurement box has the form of a cube with a side length of 60cm, representing a compromise between a volume large enough to accommodate an omnidirectional sound source and the geometric limitations resulting from the dimensions of the materials used to construct the walls, i.e., gypsum plasterboard panels. The mass of the setup in its basic configuration is approximately 18kg, which further supports its mobility.

The enclosure was designed to allow measurements on square test specimens with a side length of approximately 53cm. All walls, except for the top wall, were constructed as partitions with enhanced sound insulation. For this purpose, a three-layer structure made of high-density gypsum plasterboards was used, characterized by a weighted sound reduction index of $R_{w}\approx 55\;\mathrm{dB}$. This solution was intended to limit sound transmission through the side and bottom walls and thereby direct the dominant transmission path through the top wall of the box, which acts as the test partition.

The top wall of the enclosure was designed as a replaceable element. This solution allows different base partitions to be installed and compares the effectiveness of metamaterial linings for partitions with different initial sound insulation performance. The active area of the test partition is approximately 53×53cm, and its mounting method was kept unchanged throughout all measurement series in order to ensure comparable boundary conditions.

An important assumption in the design of the setup was the reduction in air leaks that could affect the measurement results. A cable gland was provided in the lower part of the enclosure to allow the routing of cables supplying the sound source, while the joints between the walls and the frame structure were sealed with acoustic foam tape. A view of the aluminium frame and the version enclosed with gypsum plasterboards is shown in Figure 1.

The enclosure walls were mounted using threaded rods and screws with washers, which ensured rapid assembly and disassembly while maintaining the required stability and airtightness of the joints. For the upper test partition, an additional linear clamping system using steel flat bars was applied. This solution made it possible to obtain uniform pressure along the perimeter of the panel, improved the airtightness of the joint, and limited local stress concentrations, which is important during repeated installation and removal of the partition in successive measurement series. Acoustic foam was also used at the interface between the partition and the box structure, providing sealing along the entire perimeter.

### 2.2. Instrumentation

**Figure 2:**
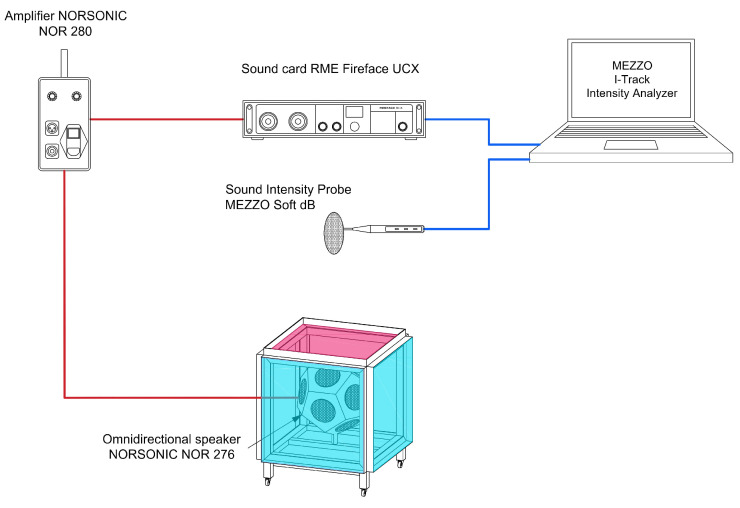
Schematic of the measurement chain. In the measurement box, the side permanent walls and permanent bottom wall are highlighted in blue, while the top replaceable wall is highlighted in pink. Red and blue lines denote transmitting and receiving path, respectively.

The measurement chain used in the study, the schematic of which is shown in Figure 2, consisted of a Nor276 omnidirectional sound source (Norsonic, Tranby, Norway), operated together with a Nor280 power amplifier and signal generator (Norsonic), an Fireface UCX USB (RME, Haimhausen, Germany) audio interface, and a computer. The measurement signals were generated in the Matlab R2023a environment, while data acquisition was carried out using the I-Track Intensity Analyzer software.

A Mezzo Soft dB sound intensity probe equipped with 40GI measurement microphones (G.R.A.S., Holte, Denmark) was used to measure the acoustic field on the receiving side. The probe was used to measure the sound intensity level over a defined measurement surface located above the test partition. To ensure repeatable positioning of the probe, a microphone stand and dedicated holders designed specifically for the setup and manufactured using 3D printing technology were used. In addition, a positioning element was employed to align the probe perpendicular to the surface of the tested panel, ensuring the required measurement geometry at successive grid points.

To ensure the comparability of the results between successive measurement series, the configuration of the entire measurement chain was kept unchanged. During the study, neither the chain components nor the instrument settings nor the gain values were modified, so all series were carried out under identical acquisition conditions. This reduced the influence of possible variations in the measurement chain parameters on the determined values of $\Delta L_{I}$.

### 2.3. Measurement Room

The experiments were conducted in one of the rooms located in the Wind Engineering Laboratory Building of Cracow University of Technology. This space was selected due to the lack of access to specialized chambers and laboratory rooms intended for standardized sound insulation measurements. At the same time, it provided an opportunity to assess the feasibility of conducting comparative measurements of the increase in sound insulation provided by metamaterial structures under typical indoor conditions.

The maximum geometric dimensions of the room were 3.3×3.6×2.7m, corresponding to a volume of approximately $31\;m^{3}$. To reduce the influence of reflections from the walls, ceiling, and floor, a partial acoustic treatment was applied, based on the principle that in each pair of parallel partitions at least one surface should be covered with sound-absorbing material. The ceiling treatment consisted of a suspended ceiling made of pyramidal polyurethane foam elements with a height of 6cm (including a 2cm base layer), mounted on a CD60 profile structure at a distance of 42cm from the ceiling slab, with a total area of $4.8\;m^{2}$. One of the side walls was covered with trapezoidal polyurethane foam elements with a thickness of 5cm, bonded directly to the substrate over an area of $5.0\;m^{2}$. The rear wall treatment was implemented using a recess filled with mineral wool with a density of $150\;\mathrm{kg}/m^{3}$ and a variable thickness of 10–20cm, covered with a single layer of acoustic nonwoven fabric, over a total area of $6.3\;m^{2}$.

During the measurements, the environmental conditions in the room were monitored. In particular, air temperature, relative humidity, and atmospheric pressure were controlled and remained stable throughout the measurement sessions.

**Figure 3:**
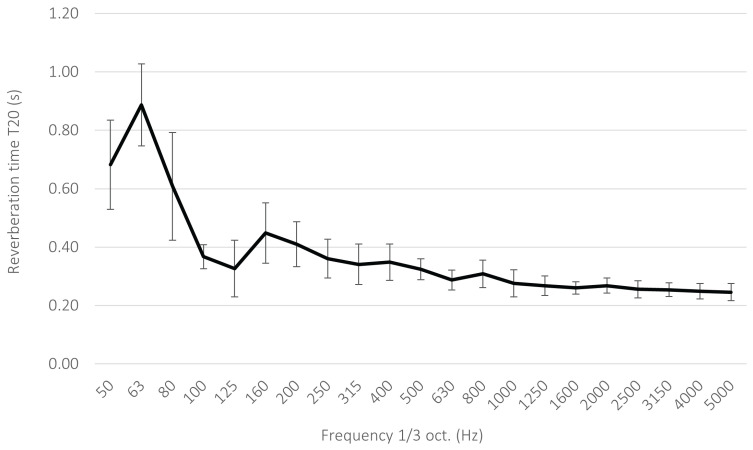
Reverberation time in the room after acoustic treatment. Error bars indicate the standard deviation obtained from the measurement points.

After the acoustic treatment was applied, a clear reduction in reverberation time was observed, with the values determined from the analysis of the recorded room impulse responses (Figure 3). In the 100–5000Hz range, the reverberation time did not exceed 0.5s, while for most of the analyzed one-third-octave bands it remained below 0.4s. These results indicate that, despite using a room not originally intended for acoustic measurements, it was possible to obtain acoustic conditions favorable for the experiments and to limit the influence of reflections on the recorded quantities.

## 3. Measurement Procedure

The measurement procedure was developed as a comparative method to determine the reduction in noise transmission through the tested partition resulting from the application of metamaterial lining. The directly determined quantity was the difference in sound intensity level, $\Delta L_{I}$, on the receiving side, defined between the reference state and the modified state. The analysis was limited to one representative partition of the enclosure, namely the upper wall, above which a single virtual measurement surface was defined. This approach reduced both the measurement and the data-processing time while maintaining comparable measurement conditions for the two states of the tested system.

In the adopted methodology, it was assumed that due to the improved sound insulation of the remaining walls of the measurement box, the main contribution to the sound intensity recorded on the receiving side originated from transmission through the tested upper partition. This assumption follows from the design of the setup, in which the side and bottom walls were constructed as highly sound-insulating partitions, and the joints between the elements were additionally sealed. At the same time, the use of a sound intensity probe and the definition of a measurement surface parallel to the tested partition made it possible to record the sound intensity associated with radiation from that surface, thereby reducing the influence of signals arriving from other directions. However, the contribution of flanking paths, including transmission through structural connections and local air leaks, was not analyzed separately and constitutes one of the limitations of the adopted method.

The tested structures were excited using an omnidirectional sound source placed inside the measurement box in a fixed position, centrally with respect to all pairs of opposite enclosure walls. The effectiveness of the applied metamaterial lining was determined on the basis of the sound intensity level recorded on the receiving side. For each frequency band, the analyzed quantity was defined as the difference in sound intensity level:

(1) $$\Delta L_{I}\left(f\right)=L_{I,\mathrm{ref}}\left(f\right)-L_{I,\mathrm{meta}}\left(f\right),$$

where $L_{I,\mathrm{ref}}\left(f\right)$ denotes the sound intensity level for the base partition, and $L_{I,\mathrm{meta}}\left(f\right)$ denotes the sound intensity level after installation of the metamaterial lining.

The quantity defined in this way is analogous to IL-type indicator, understood as the difference between the levels of energetic quantities recorded on the receiving side before and after the introduction of the tested element into the system. Unlike absolute sound insulation indicators such as TL or the laboratory sound reduction index *R*, the quantity $\Delta L_{I}\left(f\right)$ is comparative in nature and refers directly to the specific measurement setup, the system geometry, and the excitation conditions.

In the adopted methodology, the sound intensity level recorded on the receiving side was treated as a measure of the acoustic energy transmitted through the tested partition. This means that a reduction in the sound intensity level after installation of the metamaterial lining corresponds to a reduction in the acoustic energy transmitted through the partition into the receiving space. Since the geometry of the setup, the source position, the measurement surface, and the excitation conditions were kept unchanged in both states of the tested system, the difference $\Delta L_{I}\left(f\right)$ describes the change in the transmission properties of the tested partition. In this interpretation, an increase in the value of $\Delta L_{I}\left(f\right)$ indicates a greater reduction in the flow of acoustic energy to the receiving side, and therefore an improvement in the sound insulation performance of the system. For this reason, the quantity $\Delta L_{I}\left(f\right)$ was adopted as a measure of the increase in sound insulation obtained through the use of the metamaterial lining.

Data acquisition was carried out on a 3×3 grid of measurement points (9 points in total), defined on a virtual measurement surface parallel to the surface of the tested sample or test partition. The virtual surface was positioned at a distance of 10cm from the tested panel. The central point of the grid coincided with the geometric center of the sample, while the remaining points were distributed symmetrically with respect to both principal axes of the partition, with equal spacing and a constant offset from its edges. This arrangement of points was intended to provide a representative mapping of the sound intensity distribution across the entire surface of the tested partition. The distance between the virtual measurement surface and the sample was selected based on an analysis of the spatial stability of the results, while also seeking to reduce the influence of near-field phenomena associated with the partition’s natural vibrations and the excessive contribution of side reflections, which could become significant at greater distances. An additional argument for choosing this distance was the increasing uncertainty in the positioning of the measurement points as the distance from the sample increased. Importantly, the method described above of selecting fixed measurement points for measuring the sound intensity on the receiving side is consistent with the requirements of the ISO 15186-1:2000 standard [8].

At each measurement point, time averaging with a duration of at least 10s was applied. Sound intensity levels were recorded with a resolution of 10Hz over the range 100–5000Hz. For each frequency band, the difference in sound intensity level was determined separately at each of the nine measurement points according to:

(2) $$\Delta L_{I}^{\left(k\right)}\left(f\right)=L_{I,\mathrm{ref}}^{\left(k\right)}\left(f\right)-L_{I,\mathrm{meta}}^{\left(k\right)}\left(f\right),$$

where k=1,2,…,9 denotes the measurement point number, while $L_{I,\mathrm{ref}}^{\left(k\right)}\left(f\right)$ and $L_{I,\mathrm{meta}}^{\left(k\right)}\left(f\right)$ correspond to the sound intensity levels recorded at the *k*-th point for the base partition and for the partition with the installed metamaterial lining, respectively. This approach made it possible to reduce the influence of local spatial effects, including plate vibration modes that could distort the result. Next, for each frequency band, the values obtained were spatially averaged to determine a representative value:

(3) $$\bar{\Delta L_{I}}\left(f\right)=\frac{1}{9}\sum_{k=1}^{9}\Delta L_{I}^{\left(k\right)}\left(f\right).$$

The value $\bar{\Delta L_{I}}\left(f\right)$ determined in this way constituted the final measurement result for the analyzed frequency band.

The excitation conditions were kept constant between the measurement series. The level of the generated signal was calibrated at the beginning of the study so that, in the receiving room and in the most unfavorable case, i.e., for the highest sound insulation of the tested structure reinforced with the metamaterial lining, a signal-to-background-noise ratio of not less than 10dB was ensured. In addition, before each measurement series, the background noise level was analyzed in order to verify compliance with this criterion.

## 4. Verification of the Measurement Method

In order to verify the suitability of the developed measurement setup for the investigation of metamaterial structures with a sound-insulating function, to assess the applicability of the proposed comparative method and to determine whether the setup makes it possible to analyze the influence of metamaterial structures on partitions with different sound insulation performance, the following procedure was adopted. First, the structure was designed and analyzed numerically using the finite element method in the COMSOL Multiphysics 5.4 environment. This analysis was a preliminary step, used exclusively to select the geometric parameters of the system and tune it to a frequency of approximately 460Hz. Next, based on the resulting model, physical samples of the metamaterial structures were fabricated using 3D printing technology for experimental testing. In the final stage, measurements were conducted using the developed test setup on two variants of base partitions with different sound-insulating properties.

### 4.1. Design and Numerical Analysis of the Metamaterial Structures

**Figure 4:**
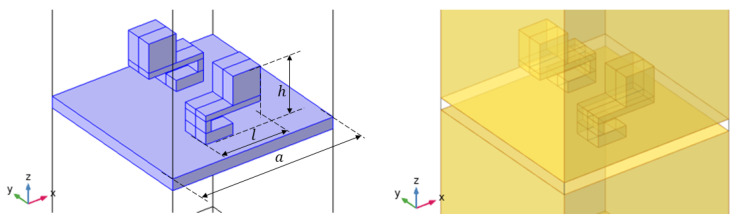
View of the numerical model of the metamaterial-structure unit cell: solid domain (in violet) on the left and air counterpart (in yellow) on the right in the COMSOL Multiphysics environment.

The design of the investigated metamaterial structure was developed using numerical analysis performed with the finite element method in the COMSOL Multiphysics environment. The adopted modeling approach and the general design concept were based on solutions described in the literature for locally resonant acoustic metamaterial structures analyzed using a representative unit-cell model [20,24]. In the numerical analysis, a unit-cell model containing two resonant elements mounted on a thin base plate was adopted. As noted in studies such as [24], this type of acoustic metamaterial generates a stop band around the mass-spring-mass resonance frequency. It has been demonstrated that the resulting attenuation significantly exceeds what can be achieved through mass increase alone. Furthermore, the frequency range of this attenuation zone can be precisely tuned via design parameters (such as the cantilever stiffness and the resonating mass), enabling effective subwavelength attenuation. A representative numerical model of the cell, developed in COMSOL Multiphysics, is shown in Figure 4.

The aim of the calculations was to select the geometric parameters of the system so as to obtain an increase in airborne sound insulation in the vicinity of 460Hz. The choice of this frequency followed from the assumptions of the research project within which the measurement setup was developed. The numerical analysis, therefore, constituted the design stage preceding the fabrication of the physical samples and their subsequent experimental investigation.

**Figure 5:**
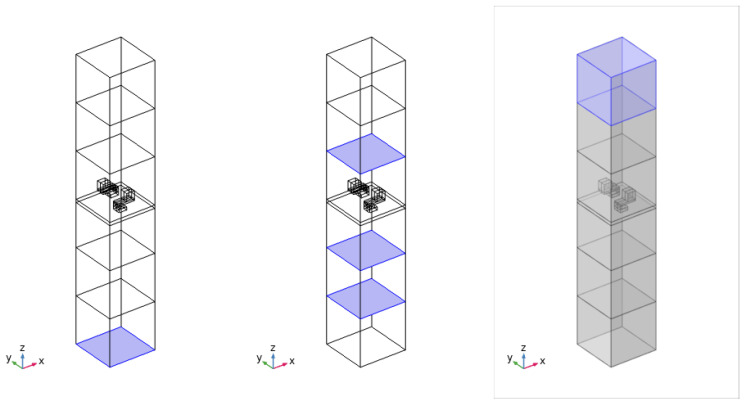
Numerical setup: from left to right (in violet), plane wave excitation surface, microphone locations, PML layer.

The numerical setup is shown in Figure 5, and the simulations were performed in the frequency domain. It should be noted that the setup mimics an impedance tube test; therefore, a direct quantitative comparison with experimental measurements cannot be drawn. However, the operating attenuation frequency can be effectively tuned and identified. No further frequency shifts are expected from changes in boundary conditions, as the attenuation mechanism is intrinsically linked to the local resonance of the internal element. The analyzed sample, i.e., a thin plate with two resonating units, was modeled as a linear elastic solid using the Solid Mechanics module and embedded in a homogeneous acoustic domain described by the Helmholtz equation using the Pressure Acoustics module. The following air properties were assumed: wave speed c=343 m/s and density $\rho =1.2\;{\text{kg/m}}^{3}$. A plane-wave excitation with unit amplitude was applied at the outer bottom boundary, while a perfectly matched layer (PML) was implemented in the upper air domain to minimize spurious reflections. Periodic boundary conditions were imposed on the lateral faces. The coupling conditions at the acoustic–solid interface were defined according to the default coupling settings. The TL coefficient was obtained using a three-point method [38], in which the averaged pressure values were calculated at the three locations indicated in Figure 5.

In the numerical model, a structural cell containing two resonant elements was analyzed. These elements were mounted on a thin base plate with a thickness of 2mm, whose role was to ensure the appropriate positioning of the metamaterial structure on the surface of the partition. At the calculation stage, the analysis was therefore focused on the resonance frequency of the metamaterial structure itself, without accounting for the target base partition used later in the experimental study. This simplification made it possible to assess the influence of the resonator geometry on the location of the characteristic frequencies and to select a configuration intended for fabrication.

The sample material was assumed to be PLA, in accordance with the manufacturing technology used to produce the physical models. The detailed material properties adopted in the numerical analysis will be given later in the paper. The basic dimensions of the analyzed structure are presented in Figure 4, where a=29.4 mm, l=20 mm, h=16.9 mm. Based on the obtained results, a configuration with a tuning frequency close to 460Hz was selected and then fabricated using 3D printing technology for further experimental investigation.

Thus, the numerical analysis served a dual purpose: a design function, related to the selection of the metamaterial-structure geometry, and a verification function, enabling a preliminary assessment of whether the designed system could be used in the experimental investigations carried out using the developed measurement setup.

### 4.2. Fabrication of Samples for Experimental Testing

**Figure 6:**
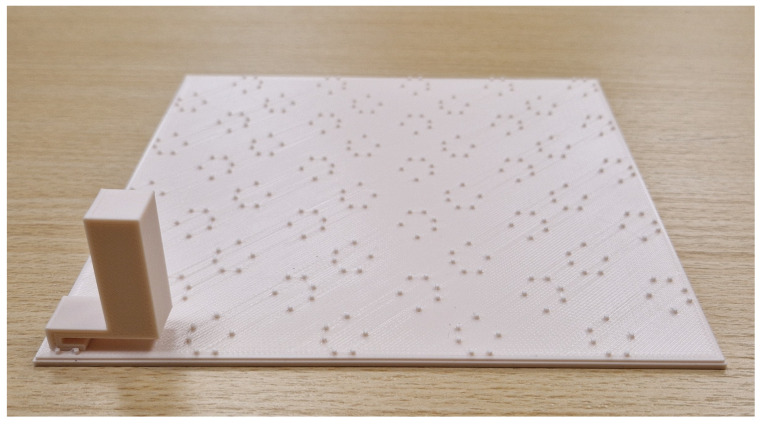
Base plate with one metamaterial element installed.

Based on the results of the numerical analyses, physical samples of the metamaterial structures were fabricated for experimental testing. The samples were manufactured from PLA using 3D printing technology, while preserving the geometry corresponding to the configuration selected at the numerical-analysis stage. Each resonant element was printed as a separate component and then attached with cyanoacrylate adhesive to a 2mm thick base plate shown in Figure 6. To ensure repeatable positioning of the elements on the sample surface, the base plates were equipped with small positioning protrusions defining the mounting locations of the individual components.

Due to the need to cover the entire tested surface measuring approximately 53×53cm, the metamaterial structure was fabricated in the form of four separate plate segments. The prepared samples were placed directly on the base partitions, without permanent attachment to their surfaces, which made it possible to easily replace the base partitions between successive measurement series. The arrangement of the metamaterial elements remained unchanged for all analyzed partition variants, and the samples covered the entire tested sound-radiating surface.

The adopted method of sample fabrication and installation ensured consistency across the design, fabrication, and experimental stages, while also enabling comparable test conditions for all analyzed system configurations.

### 4.3. Scope of the Experimental Measurements

The measurements were carried out for two variants of base partitions with different sound insulation properties. The first variant, hereinafter referred to as GK-L, was a single gypsum plasterboard panel with a thickness of 12.5mm and a surface mass of $6.5\;\mathrm{kg}/m^{2}$, characterized by a weighted sound reduction index of approximately $R_{w}\approx 25\;\mathrm{dB}$. The second variant, denoted as GK-M, consisted of a two-layer gypsum plasterboard system, with each layer having a thickness of 12.5mm and a surface mass of $9.2\;\mathrm{kg}/m^{2}$ per layer, with a sound insulation performance of approximately $R_{w}\approx 35\;\mathrm{dB}$. Pink noise was used as the measurement signal. The measurements were performed for metamaterial structures tuned to 460Hz, as previously determined in the numerical-analysis stage. For each of the analyzed base partitions, measurements were carried out both in the reference state and after installation of the metamaterial lining.

### 4.4. Results

**Figure 7:**
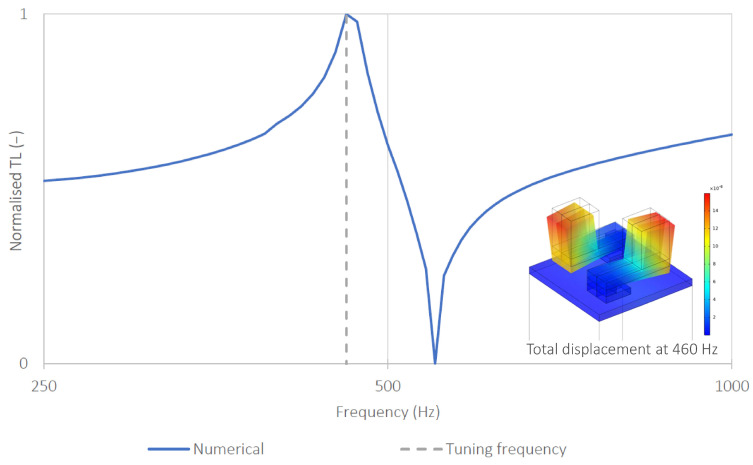
Normalized TL obtained for the tuned metamaterial sample along with the total displacement of the sample at 460 Hz. The dashed line indicates the tuning frequency of the prototype (460Hz).

Figure 7 shows the normalized TL for the tuned metamaterial. The observed TL peak corresponds precisely to the target frequency of 460 Hz. Additionally, the vibration mode at this frequency confirms the local resonance mechanism of the resonating element.

**Figure 8:**
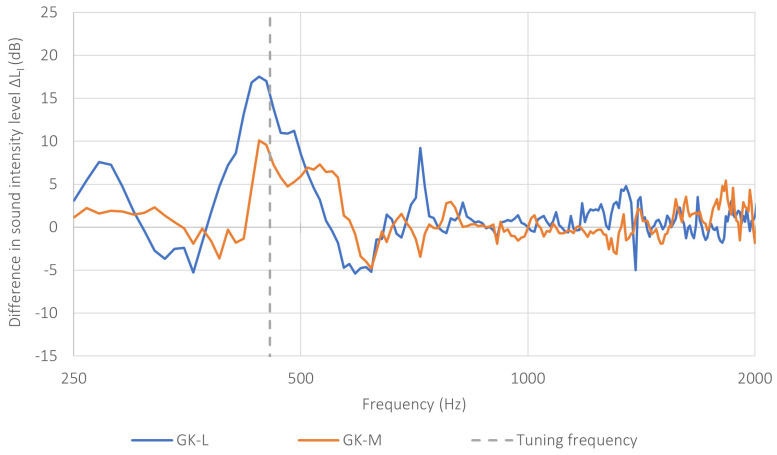
The reduction in noise transmission through the tested samples is expressed as the difference in sound intensity level $\Delta L_{I}$ for two variants of base partitions: GK-L and GK-M. For each frequency, $\Delta L_{I}$ was first determined at each of the nine measurement points and then averaged over all the points. The dashed line indicates the tuning frequency of the prototype (460Hz).

Figure 8 presents the characteristics of the reduction in noise transmission through the tested samples, expressed as the difference in sound intensity level $\Delta L_{I}$, for the two base-partition variants, GK-L and GK-M, obtained under pink-noise excitation. The tuning frequency of the metamaterial structure, equal to 460Hz, is also indicated in the figure. For the sake of clarity, the frequency range displayed was limited to 250–2000Hz, since this interval was sufficient to illustrate the structural response relevant to the effect analyzed in this study.

In both analyzed cases, a clear increase in $\Delta L_{I}$ was observed in the vicinity of the tuning frequency. This means that, in the frequency range adjacent to 460Hz, the applied lining reduced the acoustic energy transmitted through the tested partition. The maximum effect was recorded for the GK-L variant, for which $\Delta L_{I}$ reached approximately 17.5dB at 440Hz. For the GK-M variant, the increase was also clearly visible, although smaller, reaching approximately 10.1dB at the same frequency.

The characteristics indicate that the effect of the metamaterial structure was not limited to a single frequency, but extended over a broader band of approximately 430–470Hz, within which elevated values of $\Delta L_{I}$ were observed. In this range, a positive increase in $\Delta L_{I}$ was observed at all measurement points for both base-partition variants. This indicates the spatially consistent nature of the metamaterial effect and its influence over the entire surface of the tested partition.

**Figure 9:**
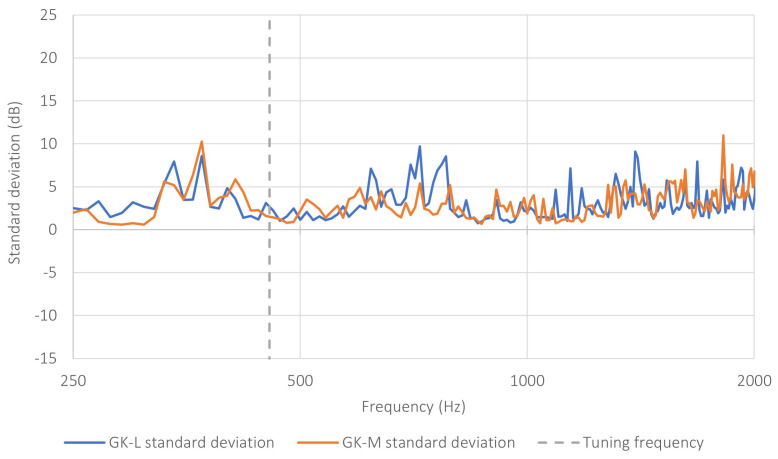
Frequency-dependent standard deviation of the difference in sound intensity level $\Delta L_{I}$, determined on the basis of the nine measurement points for the GK-L and GK-M base-partition variants. The dashed line indicates the tuning frequency of the prototype (460Hz).

Additional information on the stability of the observed effect is provided by the analysis of the spatial scatter of the results, expressed as the standard deviation determined on the basis of the nine measurement points. The frequency-dependent standard deviation for both base-partition variants is shown in Figure 9. For the GK-L variant, at the peak of the characteristic (440Hz), the standard deviation was approximately 1.18dB, whereas for the GK-M variant it was approximately 2.26dB. This means that, in the frequency range corresponding to the operation of the resonant structure, not only were the highest values of $\Delta L_{I}$ observed, but the scatter of the results was also relatively small, which confirms the stable nature of the effect. At neighboring frequencies, the scatter remained moderate, whereas outside the resonant range, it tended to increase, as also reflected in Figure 9.

The obtained results indicate that the effectiveness of the tested metamaterial structure depended on the properties of the base partition on which it was mounted. For the partition with lower sound insulation, the effect of the lining was more pronounced, whereas lower values of $\Delta L_{I}$ were observed for the partition with higher sound insulation. This may indicate that the influence of the locally resonant metamaterial structure becomes more evident in systems with lower initial performance, for which the contribution of the additional transmission-reduction mechanism is relatively greater. At the same time, the agreement between the location of the maximum $\Delta L_{I}$ value and the tuning frequency of the metamaterial structure determined at the numerical-analysis stage provides further confirmation of the validity of the adopted measurement method. This means that the proposed approach enables not only a qualitative assessment of changes in the system’s properties, but also a reliable representation of the characteristic physical phenomena associated with the operation of locally resonant structures.

Outside the mid-frequency range, the $\Delta L_{I}$ characteristics were more irregular. In the low-frequency range, local increases and decreases in the value of the indicator were observed, which may be associated with the influence of resonant phenomena in the system and with the boundary conditions of the partition. In the higher frequency ranges, the $\Delta L_{I}$ values oscillated around lower mean levels, while the scatter of local fluctuations increased, which is confirmed by the higher values of the standard deviation. Nevertheless, the principal feature of the system response, namely the occurrence of a distinct maximum in effectiveness near the tuning frequency, was clearly visible for both analyzed base-partition variants.

The results confirm that the developed measurement setup and the adopted comparative method enable the registration of changes in the system response resulting from the application of a metamaterial structure. At the same time, the observed agreement between the location of the main $\Delta L_{I}$ maximum and the designed tuning frequency indicates that the adopted design and numerical assumptions were qualitatively confirmed in the experimental measurements.

Finally, it’s worth noting that the study presented in this chapter was repeated several times to confirm the stability of the obtained results. Each time, the entire sample assembly and measurement chain procedure was repeated. The variation between conducted measurements in individual frequency bands typically did not exceed 2 dB.

## 5. Summary and Conclusions

This paper presented an original method for determining the reduction in noise transmission through the tested partition provided by small samples of metamaterials, based on comparative sound intensity measurements. The developed test setup, in the form of a mobile, modular measurement box, enables investigations under near-real conditions while reducing requirements for specimen size and laboratory infrastructure. The proposed approach constitutes a practical alternative to classical methods that require large specimens, specialized chambers, and time-consuming installation procedures.

The adopted methodology makes it possible to assess the effectiveness of metamaterial linings on the basis of the difference in sound intensity level, $\Delta L_{I}$, interpreted as a measure of the increase in sound insulation of the tested partition. The use enables assessment of the effectiveness of metamaterial linings based on this quantity, which enabled a direct comparison of the system response in the reference state and after installation of the metamaterial structure, while maintaining unchanged measurement conditions.

The experimental investigations confirmed the usefulness of both the developed setup and the measurement method itself. In both analyzed cases, a clear noise transmission reduction was observed in the vicinity of the tuning frequency of the metamaterial structure, equal to approximately 460 Hz. The maximum effect was obtained for the partition with lower sound insulation, for which the value of $\Delta L_{I}$ reached approximately 17 dB, whereas for the partition with higher sound insulation, the maximum increase was approximately 10 dB. The obtained results indicate that the effectiveness of the locally resonant metamaterial structure depends on the properties of the base partition and is pronounced in systems with lower initial sound insulation.

Particular attention should also be paid to the qualitative agreement between the experimental results and the design assumptions adopted at the numerical-analysis stage. The location of the main maximum of the $\Delta L_{I}$ characteristic near the tuning frequency confirms that the designed metamaterial structure operates in accordance with the expected local-resonance mechanism and can effectively reduce the transmission of acoustic energy within the selected frequency band. Unfortunately, it was not possible to compare the measured results of $\Delta L_{I}$ index with numerical simulations due to the excessive complexity of the FEM model and the lack of computational power of available computers for this type of simulation.

The developed method is comparative in nature, which is an important advantage in prototype investigations, since it enables the assessment of the influence of the applied structure on changes in the acoustic properties of the partition in a relatively simple and rapid manner. At the same time, it should be noted that the obtained results may also be affected by the characteristics of the measurement system itself, such as the boundary conditions, the contribution of flanking transmission, or local effects related to the geometry of the setup. This does not change the fact, however, that the method proved sufficiently sensitive to capture the essential differences between the analyzed system variants and to register the effect of the metamaterial lining.

In summary, the presented setup and measurement procedure constitute a useful tool for the rapid, relatively low-cost, and reliable evaluation of the noise transmission reduction provided by small samples of metamaterials. The obtained results confirm that the proposed solution can be successfully used for comparative investigations of metamaterial structures mounted on lightweight partitions, especially in situations where setup flexibility, simplicity of installation, and the possibility of prototype testing under near-real conditions are important. It would, of course, be valuable to conduct some comparative studies on a full-scale test stand, i.e., in large coupled reverberation and anechoic chambers. This is earmarked for future work.

## Data Availability

All data are include in paper.
